# Bilateral Neuroendocrine Carcinoma of Breast: A Case Report

**DOI:** 10.31729/jnma.6997

**Published:** 2021-12-31

**Authors:** Sulochana Neupane, Sanam Dhakal, Shripad Walawalakar, Surya Bahadur Parajuli, Sulav Sapkota

**Affiliations:** 1 Birat Medical College Teaching Hospital, Biratnagar, Morang, Nepal; 2 Department of Pathology, Biratnagar Hospital Pvt. Ltd., Biratnagar, Morang, Nepal; 3 Department of Community Medicine, Birat Medical College and Teaching Hospital, Biratnagar, Morang, Nepal; 4 Department of Medical Oncology, Birat Medical College Teaching Hospital, Biratnagar, Morang, Nepal

**Keywords:** *adjuvant chemotherapy*, *breast*, *neoplasms*, *neuroendocrine tumor*

## Abstract

Primary neuroendocrine carcinomas of the breast are rare of all breast carcinomas. They may be well-differentiated, poorly differentiated, or invasive breast cancers with neuroendocrine differentiation. They are staged and treated similarly to conventional breast cancer. Herein, we report a case of invasive ductal carcinoma with neuroendocrine differentiation of the breast in a 73 years female with a history of breast lump initially in the lower inner quadrant of left breast and a month later, similar lump at the same site in right breast. Patient underwent Modified Radical Mastectomy bilaterally followed by adjuvant chemotherapy based on Carboplatin and Etoposide regimen.

## INTRODUCTION

Primary neuroendocrine tumors of the breast are the rare breast cancers. Neuroendocrine differentiation in breast carcinomas was first described by Feyrter and Hartmannin 1963, based on positive silver staining in mucinous carcinomas of the breast.^1^ These tumors occur predominately in postmenopausal women in sixth to seventh decades. According to the World Health Organization (WHO), these are subclassified into three groups: Well-differentiated neuroendocrine tumors (WD-NET), Poorly differentiated neuroendocrine tumors (PD-NET) or small cell carcinoma and Invasive breast carcinoma with neuroendocrine differentiation (IBC-NED). The diagnosis requires expression of neuroendocrine markers synaptophysin and chromogranin A. We present a 73 years-old-female with a history of breast lump six months back initially in the left breast, about the size of a peanut.

## CASE REPORT

A 73 years-old-female present with a history of breast lump six months back initially in the left breast, in the lower inner quadrant about the size of a peanut. The lump was at first detected accidentally because of pain while she was handling her grandchildren. About a month later, she had a similar lump, almost the same size in the same site of her right breast as in the left. The lump grew to the size of an almon d as time progressed and they began to be more tender and immobile. Both lumps were fixed to the skin and underlying breast tissues.

On examination, a single palpable lump was in the lower inner quadrant in each breast. Skin over the lump was normal. There was no dimpling and venous engorgement, no peau d' orange appearance, no fissuring, fungating and eczema seen. Nipples were normally located bilaterally. Areola were normal bilaterally. The temperature over both breasts was normal. Both lumps were located in the lower inner quadrant which were of size around 1cmx0.5cm which were firm to hard in consistency, tender, with well-defined margin and smooth surface, immobile and fixed to the skin and underlying breast tissue. There was no nipple discharge on squeezing. There was bilateral involvement of level I and II axillary nodes. She also complained of decreased appetite, weight loss and generalized body weakness.

**Figure 1:**
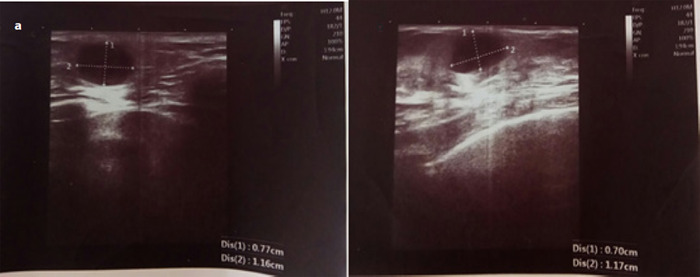
(a) Ultrasonography of the right breast, (b) Left Breast.

Ultrasonography revealed lumps in both breasts at six o'clock position which were well defined, hypoechoic, nodular and oval measuring 11.6x7.7mm (right breast) (Figure 1a) and 11.7x7mm (left breast) (Figure 1b).

**Figure 2:**
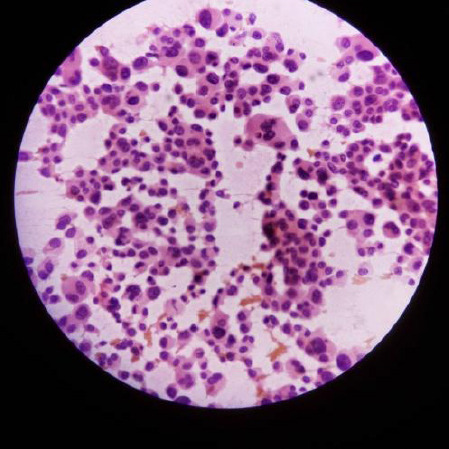
FNAC of enlarged axillary lymph nodes in high power microscopy (40X).

Fine Needle Aspiration Cytology (FNAC) done from each breast lump revealed an impression compatible with neuroendocrine tumor. FNAC from enlarged axillary nodes showed tumor cells scattered singly as well as in sheets Fine Needle Aspiration Cytology (FNAC) done from each breast lump revealed an impression compatible with neuroendocrine tumor. FNAC from enlarged axillary nodes showed tumor cells scattered singly as well as in sand clusters with moderate cellular pleomorphism, hyperchromatic nuclei with inconspicuous nucleoli and scanty cytoplasm. Few bizarre shaped cells and multinucleated cells were also seen with hemorrhagic background suggestive of metastatic carcinoma (Figure 2).

**Figure 3:**
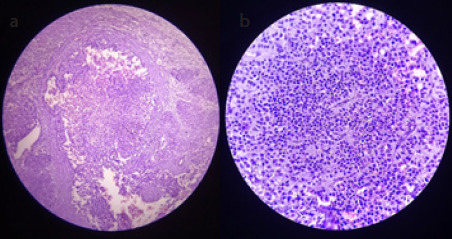
(a) Histopathological section of excisional biopsy material of breast in low-power microscopy (10X), (b) High-power microscopy (40X).

Carcinomas of both breasts were of stage IIIA (T1N2M0). Surgery was planned and she underwent Modified Radical Mastectomy. Biopsy of the excised breast lump was done. The histopathological section showed tumor cells arranged in sheets separated by fibrous tissue. Cells had oval to rounded nucleus and abundant cytoplasm with minimal nuclear polymorphism suggestive of Invasive ductal carcinoma with neuroendocrine differentiation (Figure 3a, 3b).

The most common two confusing varieties of breast carcinoma in comparison with our case were ductal carcinoma in-situ and invasive ductal carcinoma. These were excluded after histopathological examination of tissue excised.

The patient underwent Modified Radical Mastectomy. About 21 days after the surgery, adjuvant chemotherapy based on Carboplatin and Etoposide was started.

The patient is improving. There has been a plan to reevaluate the case, discuss in multidisciplinary meetings and plan for further management.

## DISCUSSION

Neuroendocrine tumors are malignant tumors derived from neuroendocrine cells. They most commonly occur in the gastrointestinal tract (48%), lung (25%) and pancreas (9%), but may also develop in many other organs, including the breast, prostate, thymus and skin.^2^ Neuroendocrine cells have the capability to produce hormones, such as serotonin, which can result in symptoms such as flushing and diarrhea, as well as other proteins (e.g. chromogranin A), which serve as biomarkers.^2^ The neuroendocrine tumors of breasts are very rare tumors. Histopathological examination along with immunohistochemistry is the mainstay of diagnosis. Histopathologically the tumor cells are arranged in sheets, ribbons and cords. Individual cells are uniform with round to ovoid nucleus, salt and pepper chromatin and scant cytoplasm.^3^ Immunohistochemistry shows more than 50% tumor cells positive for neuroendocrine tumor markers like chromogranin, synaptophysin and NSE (Neuron-Specific-Enolase).^4^ Immunohistochemistry is not available in our hospital setting and the diagnosis was based purely on histopathological examination of the excised breast tissue.

We are the first to report this rare clinical entity from Nepal. In a case report from India, a 67-year postmenopausal patient with primary neuroendocrine tumor of breast was treated with adjuvant chemotherapy, adjuvant radiotherapy and was started on hormonal therapy with tamoxifen (20mg/day).^5^ Our case also reports a similar aged (73 years) post-menopausal woman responsive to the adjuvant chemotherapy.

Also, a similar case from Malaysia, was started with a combination of cisplatin and etoposide (100mg/m^2^) neoadjuvant chemotherapy based on the protocol for small cell lung carcinoma.^6^ In our case, the patient underwent Modified Radical Mastectomy bilaterally followed by adjuvant chemotherapy based on carboplatin and etoposide regimen.

Lacking adequate study and research in the field of these tumors, there are no standard treatment protocols and they have been treated like other invasive carcinomas. The prognosis about these tumors is not known exactly. Though neuroendocrine tumors of the breast are a rare, they must be a kept as differential of breast lump in postmenopausal period. Immunohistochemistry is the gold standard diagnostic modality. Surgery followed by adjuvant chemotherapy shows better outcomes than single therapy.
